# Combined Vitamin D and Resveratrol Treatment Attenuates Motor Deficits and Neurodegeneration in a 6‐OHDA Mouse Model of Parkinson's Disease in Male Mice: A Focus on Sirt1 and Lingo‐1 Pathways in the Ventral Tegmental Area

**DOI:** 10.1002/brb3.71673

**Published:** 2026-08-17

**Authors:** Monavareh Soti, Mehran Ilaghi, Kristi A. Kohlmeier, Erfan Shahabinejad, Zeynab Pirmoradi, Mohammad Shabani

**Affiliations:** ^1^ Neuroscience Research Center, Institute of Neuropharmacology Kerman University of Medical Sciences Kerman Iran; ^2^ Department of Drug Design and Pharmacology, Faculty of Health Sciences University of Copenhagen Copenhagen Denmark; ^3^ Student Research Committee Rafsanjan University of Medical Sciences Rafsanjan Iran; ^4^ USERN Office Rafsanjan University of Medical Sciences Rafsanjan Iran; ^5^ Neuroscience Research Center Torbat Heydariyeh University of Medical Sciences Torbat Heydariyeh Iran

**Keywords:** 6‐hydroxydopamine, Lingo‐1, Parkinson's disease, resveratrol, Sirt1, vitamin D3

## Abstract

**Background:**

Parkinson's disease (PD) is characterized by progressive dopaminergic neurodegeneration, leading to severe motor impairments. *Lingo‐1* negatively regulates neuronal survival, while *Sirtuin‐1 (Sirt1)* supports mitochondrial function and oxidative stress resistance. Given the neuroprotective properties of Vitamin D3 (VitD3) and resveratrol, this study examined whether their combined administration could modulate *Sirt1* and *Lingo‐1* expression and attenuate neurodegeneration in a 6‐hydroxydopamine (6‐OHDA) model of PD.

**Methods:**

Male Swiss mice were assigned to six groups: Control, Sham, 6‐OHDA, 6‐OHDA + Resveratrol (5 mg/kg), 6‐OHDA + VitD3 (0.1 µg/kg), and 6‐OHDA + Res + VitD3. Behavioral performance was evaluated using open field, footprint, wire grip, and rotarod tests. Neuronal integrity within the ventral tegmental area (VTA) was assessed via Nissl staining, while *Sirt1* and *Lingo‐1* gene expression levels were quantified by RT‐qPCR.

**Results:**

6‐OHDA lesioning produced marked reductions in locomotor activity, grip strength, and rotarod performance, accompanied by significant neuronal loss in the VTA and dysregulated *Sirt1/Lingo‐1* expression. Treatment with VitD3 or resveratrol alone partially improved motor performance and reduced neuronal loss, whereas the combination therapy produced the most pronounced neuroprotection. Co‐administration of VitD3 and resveratrol normalized *Sirt1* expression, suppressed *Lingo‐1* upregulation, and preserved neuronal morphology in the VTA.

**Conclusions:**

Combined treatment with VitD3 and resveratrol mitigates motor deficits and neurodegeneration in 6‐OHDA‐lesioned mice which was associated with activation of *Sirt1* and suppression of *Lingo‐1*. The results suggest the therapeutic potential of targeting the *Sirt1* and *Lingo‐1* signaling pathways via nutraceutical interventions as a disease‐modifying strategy in PD.

## Graphical abstract

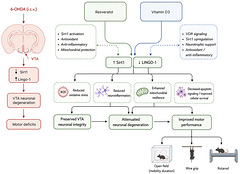

## 1 Introduction

Parkinson's disease (PD) affects over 11.7 million individuals worldwide and ranks as the second most prevalent neurodegenerative disorder (Luo et al. 2025). The pathological feature of PD includes degeneration of dopaminergic neurons in the substantia nigra pars compacta, which manifests clinically through debilitating motor symptoms such as tremor, bradykinesia, rigidity, and postural instability (Zaman et al. 2021; Marino et al. 2020). Although dopaminergic replacement therapies provide symptomatic relief, they generally fail to halt neurodegeneration and often produce adverse effects with long‐term use. Therefore, identifying neuroprotective agents capable of preserving dopaminergic function and modifying disease progression remains a critical goal in PD research.

Recent molecular investigations have identified leucine‐rich repeat and immunoglobulin‐like domain‐containing Nogo receptor‐interacting protein 1 (*Lingo‐1*) as a critical negative regulator of neuronal survival and regeneration (Kalafatakis et al. 2023). Elevated *Lingo‐1* expression has been implicated in the pathophysiology of PD and related movement disorders, where it contributes to axonal degeneration and impaired neuroplasticity (Inoue et al. 2007; Kuo et al. 2013; Pirmoradi et al. 2025). These findings suggest that therapeutic targeting of *Lingo‐1* to inhibit its signaling represents a promising approach to promote axonal repair and enhance neuronal survival in neurodegenerative conditions (Mi et al. 2013).

Another major regulator of neuronal resilience is *Sirtuin‐1 (Sirt1)*, a nicotinamide adenine dinucleotide (NAD^+^)‐dependent deacetylase that governs mitochondrial biogenesis, oxidative stress response, and anti‐apoptotic signaling (Ou et al. 2014; Merksamer et al. 2013). Reduced *Sirt1* expression or activity has been consistently documented in experimental PD models and PD patients, correlating with heightened vulnerability of dopaminergic neurons to toxic insults (Li et al. 2020; Singh et al. 2017; Zhu et al. 2021). Given its multifaceted cytoprotective functions, pharmacological activation of *Sirt1* represents a rational therapeutic strategy for attenuating neurodegeneration and promoting functional recovery in PD.

Resveratrol, a polyphenolic compound abundant in grapes and berries, is one of the best‐known *Sirt1* activators; through direct enhancement of *Sirt1* deacetylase activity, which provides antioxidant and anti‐inflammatory benefits (Rogina and Tissenbaum 2024; de Sá Coutinho et al. 2018). *Sirt1* expression is also upregulated by 1,25‐dihydroxyvitamin D3 (VitD3), a steroid with diverse neuroprotective properties including upregulation of neurotrophic factors, modulation of inflammatory responses, and enhancement of antioxidant defense systems (Shi et al. 2024). Interestingly, VitD3 deficiency is linked to increased PD risk and in preclinical studies, VitD3 supplementation protects dopaminergic neurons in toxin‐based experimental models (Shi et al. 2024; Bayo‐Olugbami et al. 2022; Lima et al. 2018). Taken together, resveratrol and VitD3 could synergize at the level of *Sirt1*, leading to enhanced cellular resilience and neuronal survival. Further, we have shown that resveratrol and VitD3 co‐administration result in downregulation of *Lingo‐1*, which could indirectly result in enhancement of neuronal repair (Pirmoradi et al. 2024).

We have previously reported that co‐administration of resveratrol and VitD3 downregulated *Lingo‐1* expression, upregulated *Sirt1*, and improved motor coordination in an experimental model of essential tremor (Pirmoradi et al. 2024). While both resveratrol and VitD3 have shown promising neuroprotective effects in several models of motor impairment, the molecular mechanisms underlying their neuroprotective effects, particularly their potential interactions with *Lingo‐1* and *Sirt1* signaling, remain incompletely characterized in a PD relevant model. Moreover, whether the combined administration of these agents might provide synergistic behavioral, molecular and cellular benefits superior to monotherapy has not been investigated in a model exhibiting dopamine cell degeneration. The present study was therefore designed to address these gaps by elucidating whether VitD3 and resveratrol, both individually and in combination, could mediate neuroprotective effects associated with alterations in *Sirt1* and *Lingo‐1* expression in a 6‐hydroxydopamine (6‐OHDA) mouse model of PD. For evaluation of effects on *Sirt1*, *Lingo‐1* and neuroprotection, we focused on the ventral tegmental area (VTA), a dopaminergic nucleus crucial for voluntary movement and motor coordination through projections to the basal ganglia and cortical motor regions (Yetnikoff et al. 2014; Hosp et al. 2019; Hassanshahi et al. 2025). VTA degeneration exacerbates motor deficits in PD models (Harrison et al. 2016; Alberico et al. 2015), highlighting its importance beyond the classical nigrostriatal pathway. Thus, preserving dopaminergic integrity within the VTA is essential for maintaining coordinated motor output and may represent an additional therapeutic neuroprotection target in PD. Accordingly, this study aims to explore the potential of combined VitD3 and resveratrol supplementation as a novel nutraceutical strategy to modulate neuroprotective pathways relevant to PD.

## 2 Methods

### 2.1 Animals

Male Swiss mice (each weighing 25–35 g) were purchased from the animal facility at Kerman University of Medical Sciences. Housing conditions were standardized, with animals maintained in groups of four per cage under a 12‐h light‐dark cycle at an ambient temperature of 22 ± 2°C. Food and water were provided without restriction throughout the experimental period. The study protocol received approval from Torbat Heydariyeh University of Medical Sciences (Ethics approval code: IR.THUMS.AEC.1405.001).

### 2.2 Groups and Experimental Design

Mice were acclimated to standardized housing conditions for two weeks prior to the initiation of experiments. During this habituation period, animals were handled daily to reduce stress and enhance their suitability for subsequent behavioral assessments.

**FIGURE 1:**
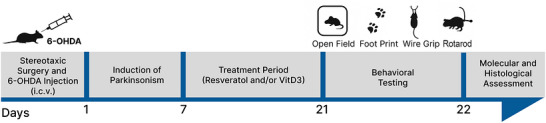
**An overview of study timeline**. Mice underwent stereotaxic surgery and intracerebroventricular injection of 6‐OHDA. Resveratrol and/or VitD3 treatments were administered intraperitoneally for 14 days. Behavioral assessments were performed before tissue collection, followed by VTA dissection for RT‐qPCR analysis and histological evaluation.

Male mice were randomly assigned to six experimental groups (*n* = 8/group) as follows: (1) Control: no surgical intervention or drug administration; (2) Sham: underwent stereotaxic surgery and received an intracerebroventricular (i.c.v.) injection of 3 µL sterile saline; (3) 6‐OHDA: Mice received an i.c.v. injection of 6‐hydroxydopamine (3 µL 6‐OHDA, 40 µg/µL in saline containing 0.05% ascorbic acid to prevent oxidation); (4) 6‐OHDA + Resveratrol: received 6‐OHDA as above, followed by intraperitoneal (i.p.) administration of resveratrol ((5 mg/kg/day; Solarbio Life Sciences, Beijing, China; Cat. No. R8350; dissolved in 10% ethanol) for 14 consecutive days; (5) 6‐OHDA + VitD3: received 6‐OHDA as above, followed by i.p. administration of VitD3 (0.1 µg/kg/day; HealthAid Ltd., UK; Health Certificate No. 5019781010424; dissolved in 10% ethanol) for 14 consecutive days; and (6) 6‐OHDA + Resveratrol + VitD3: Mice received 6‐OHDA and concurrent daily i.p. administration of both resveratrol (5 mg/kg) and VitD3 (0.1 µg/kg) for 14 days. The doses of Resveratrol and VitD3 were selected based on previous experimental studies demonstrating behavioral and neuroprotective effects of these regimens in experimental models (Pirmoradi et al. 2024; Jamhiri et al. 2025; Cai et al. 2022). The behavioral tests and molecular and histological assessments were done on days 20 and 21 of the study. The study design is depicted in Figure 1.

### 2.3 Stereotaxic Surgery and Microinjection

In this study, we employed a PD mouse model established through bilateral i.c.v. administration of 6‐OHDA (Simola et al. 2007). A single i.c.v. injection of 6‐OHDA into the lateral ventricles induces widespread degeneration of dopaminergic neurons. To inhibit noradrenergic reuptake, desipramine (25 mg/kg, i.p.) was administered 30 min before 6‐OHDA (hydrochloride salt; Sigma, USA). Mice were anesthetized with ketamine (90 mg/kg) and xylazine (10 mg/kg) via i.p. injection. A stainless‐steel guide cannula was implanted stereotaxically into the lateral ventricle using coordinates referenced to bregma: anteroposterior: 0.3 mm; mediolateral: ±1.0 mm; and dorsoventral: 2.5 mm (Shabani et al. 2023). The cannula was fixed to the skull using dental acrylic cement. Subsequently, 6‐OHDA was administered using a 25‐µL Hamilton microsyringe (Hamilton Company, Reno, NV, USA) connected to a 27‐gauge injection needle via polyethylene tubing. The solution was injected manually at a slow, controlled rate of approximately 0.5 µL/min, and the needle was left in place for 5 min before being slowly withdrawn to minimize reflux along the injection tract. Following surgery, mice were provided a 1‐week recovery period to allow stabilization of neurochemical status, ensuring comparability to vehicle‐operated controls. After the recovery phase, and once Parkinsonian‐like symptoms were evident, subsequent experimental procedures were initiated.

### 2.4 Behavioral Tests

#### 2.4.1 Open Field Test

Locomotor activity was assessed using a Plexiglas open field arena (90 × 90 × 45 cm). At the start of each session, individual mice were placed in the center, and their behavior was video‐recorded for 5 min (Ethovision 7.1, Noldus Information Technology, Netherlands). Quantitative measures included the total distance moved (TDM) and the duration of mobility (Gould et al. 2009). The apparatus was disinfected with 70% ethanol between sessions to minimize olfactory cues.

#### 2.4.2 Footprint Analysis

Gait dynamics and ambulatory patterns were assessed via footprint analysis. The plantar surfaces of the mouse hind paw were coated with non‐toxic ink, after which the animal transversed a Plexiglas corridor (100 × 10 × 10 mm) toward a darkened goal chamber, depositing inked impressions on white absorbent paper lining the runway floor. Three gait parameters were measured: stride width, right hind paw stride length, and left hind paw stride length (all expressed in centimeters). For each mouse, values were calculated by averaging three consecutive footfall sequences, excluding initial and terminal steps. Stride length was defined as the anterior‐posterior distance between successive ipsilateral paw placements, while stride width represented the mediolateral distance between contralateral hind paw placements (Soti et al. 2025).

#### 2.4.3 Wire Grip Test

To assess muscular strength and equilibrium, mice performed the wire grip test. Animals were suspended from a horizontal steel wire (80 cm in length, 7 mm in diameter), positioned 50 cm above a padded surface to prevent injury. Three trials were conducted per animal, each separated by a 5‐min interval. The latency to fall was recorded with a stopwatch as an index of grip strength and coordination (Ranjbar et al. 2022).

#### 2.4.4 Rotarod Performance

Motor coordination and balance were assessed with an accelerating rotarod device (Hugo Sachs Electronik, Germany). Animals received one day of pre‐test adaptation. During testing, each mouse was placed on a rotating rod with speeds escalating from 10 to 60 revolutions per minute. Performance was measured as the average latency to fall across three trials, with a maximum duration of 300 s and at least 5 min of rest between trials (Ranjbar et al. 2022).

### 2.5 Brain Tissue Collection and Processing

Following completion of all behavioral evaluations, animals were deeply anesthetized with isoflurane 5% and rapidly euthanized by decapitation. Brain tissue was rapidly extracted, and the VTA was gently isolated on ice, rapidly snap‐frozen in liquid nitrogen, and then maintained at −80°C until further analysis. These samples were later used for analysis of *Lingo‐1* and *Sirt1* expression levels.

### 2.6 Reverse Transcription‐Quantitative Polymerase Chain Reaction (RT‐qPCR)

#### 2.6.1 RNA Extraction and cDNA Synthesis

Total RNA was isolated from tissue specimens using Trizol reagent (Zaver Zist Azma, Iran) in accordance with the manufacturer's instructions. The concentration and purity of the isolated RNA were determined spectrophotometrically using a Nanodrop device (Thermo Scientific, USA); samples with A260/A280 ratios between 1.8 and 2.0 were deemed suitable for subsequent processing. Integrity of RNA was confirmed by electrophoresis on a 1% agarose gel. First‐strand cDNA synthesis was accomplished using the Easy cDNA Synthesis Kit (Pars tous, Iran), following the supplier's protocol, to provide templates for quantitative analysis.

#### 2.6.2 QPCR Analysis

Quantitative real‐time PCR (RT‐qPCR) was conducted utilizing SYBR Green chemistry on a Qiagen detection system (Qiagen, Germany) to quantify mRNA expression levels of *Lingo‐1* and *Sirt1*. Amplification reactions were prepared using Real Q Plus 2× Master Mix (Pars tous, Iran) and gene‐specific primers, as follows: *Lingo‐1* (Forward: 5′‐GCT GAC GCT GGA GAA ATG‐3′; Reverse: 5′‐GAA GGA GTA GTC CCG TAT GG‐3′), *Sirt1* (Forward: 5′‐GGT TGC AGG ATA AAT CCT GC‐3′; Reverse: 5′‐CCA GCC ACA AGC TGT TAA CC‐3′), and GAPDH *(reference gene*, Forward: 5′‐GCA AGT TCA ACG GCA CAG‐3′; Reverse: 5′‐GCC AGT AGA CTC CAC GAC AT‐3′). The PCR program began with an initial denaturation step at 95°C for 10 min, followed by 40 amplification cycles of 95°C for 15 s and 60°C for 60 s. Each qPCR assay was run in triplicate, with non‐template controls included to verify that no contamination was present. Relative gene expression was determined using the 2^−ΔΔCt^ method, normalizing target gene expression to that of GAPDH.

### 2.7 Nissl Staining and Quantitative Analysis of VTA Neuronal Morphology

To assess neuronal degeneration in the VTA, standard Nissl staining procedures were employed. Briefly, paraffin embedded brain tissues containing the VTA were sectioned at 5 µm thickness using a semi‐automated microtome (Leica RM2145). For histological evaluation, sections were mounted and stained with 0.1% cresyl violet (Nissl stain). To ensure consistency and rigor, four animals per experimental group (*n* = 4/group) were analyzed. For each animal, the percentage of neuronal degeneration was determined in four distinct subregions of every VTA coronal section. Microscopic examination was performed independently by two blinded investigators using a Nikon 50i Plus microscope. High‐resolution digital images were acquired with a Nikon Axiocam camera to facilitate subsequent quantification. In Nissl‐stained sections, viable neurons were identified by their clear cytoplasm, well‐defined nucleus, and prominent nucleolus, whereas degenerated neurons displayed shrunken nuclei and darkly stained (hyperchromatic) cytoplasm. Quantitative analysis of neuronal degeneration was performed by direct cell counting. In each sampled region, degenerated and healthy neurons were counted in the acquired microscopic fields. The percentage of neuronal degeneration was calculated as follows:

Percentage of degeneration = [(Number of degenerated cells / Total number of cells) × 100]. To ensure reliability, counts from both investigators were averaged. Group comparisons were performed based on mean percentage degeneration per animal. It should be noted that Nissl staining was used to assess general neuronal morphology and degeneration within the VTA and does not specifically identify dopaminergic neurons. Therefore, the histological findings were interpreted as evidence of preserved VTA neuronal integrity rather than direct quantification of TH‐positive dopaminergic neuronal survival.

### 2.8 Statistical Analysis

Statistical analyses were conducted using GraphPad Prism version 8. Results were presented as mean ± standard error of the mean (SEM). Data distribution was evaluated with the Shapiro–Wilk normality test. For data meeting normality assumptions, differences among experimental groups were analyzed by one‐way analysis of variance (ANOVA), followed by Tukey's post hoc test for multiple comparisons. Non‐parametric data were analyzed using the Kruskal–Wallis test followed by Dunn's post hoc correction. A *p* < 0.05 was applied throughout all analyses.

## 3 Results

### 3.1 Effects of VitD3 and Resveratrol Treatment on Locomotor Activity in 6‐OHDA–Lesioned Mice

**FIGURE 2:**
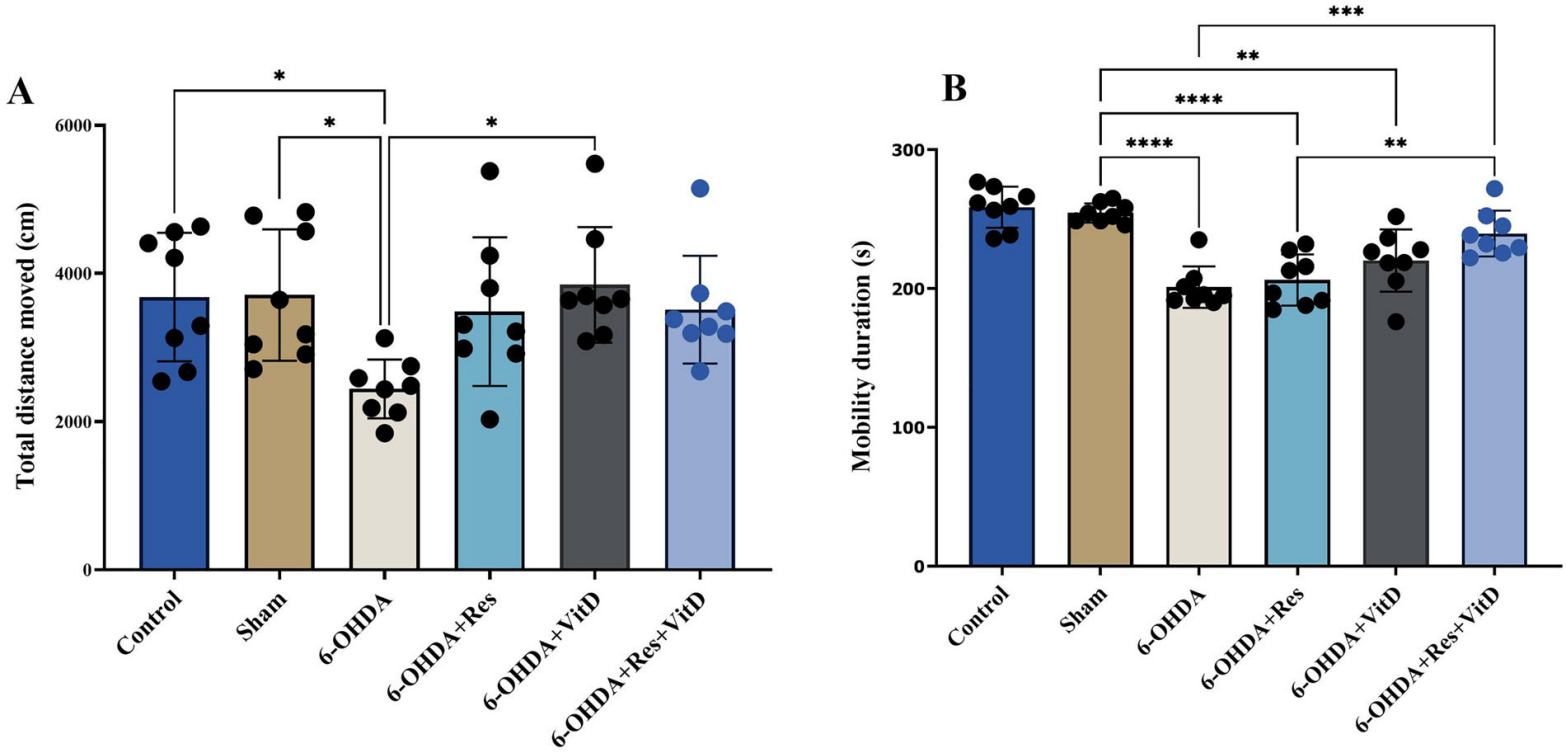
**Open field assessment of locomotor activity**. (A) Total distance moved and (B) mobility duration. Pairwise comparisons with the control group were omitted from panel B to maintain visual clarity and avoid redundancy. Data are expressed as mean ± SEM; *n* = 8 animals per group. Statistical comparisons were performed using one‐way ANOVA followed by Tukey's post hoc test. *p < 0.05, **p < 0.01, ***p < 0.001, ****p < 0.0001.

One‐way ANOVA demonstrated a significant main effect of treatment on TDM in the open field test (*F*(5, 42) = 3.25, *p* < 0.05). Pairwise comparisons indicated that the TDM was significantly decreased in the 6‐OHDA group compared to both the control and sham groups (*p* < 0.05), highlighting locomotor impairment induced by 6‐OHDA. Treatment with VitD3 significantly increased the TDM compared to the untreated 6‐OHDA group (*p* < 0.05). Although the combined treatment of resveratrol and VitD3 resulted in enhancement of TDM, and approached values observed in the sham and control groups, the difference was not statistically significant compared to the 6‐OHDA group (Figure 2A).

A robust main effect of treatment was observed on the mobility duration in the open field test (*F*(5, 42) = 17.99). The 6‐OHDA group exhibited a marked reduction in mobility time compared to both control and sham groups (*p* < 0.0001), confirming substantial locomotor impairment following dopaminergic lesioning. Notably, co‐administration of resveratrol and VitD3 led to a statistically significant restoration of mobility compared to 6‐OHDA alone (*p* < 0.001). However, these effects were not observed in monotherapies (Figure 2B).

### 3.2 Gait Parameters Remain Unchanged Following 6‐OHDA Lesion and Interventions

**FIGURE 3:**
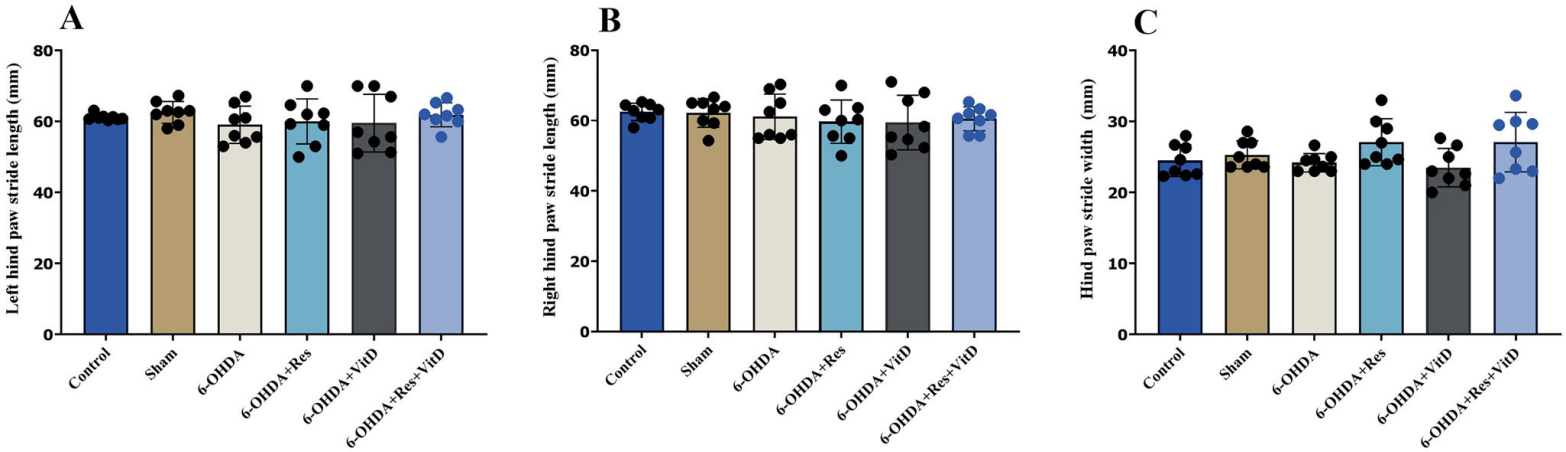
**Analysis of gait pattern using footprint test**. (A) Left hind paw stride length, (B) right hindpaw stride length, and (C) hind paw stride width measurements. Data are expressed as mean ± SEM; *n* = 8 animals per group. Statistical comparisons were performed using one‐way ANOVA.

Footprint analysis was employed to objectively assess gait and coordination in experimental groups. One‐way ANOVA revealed no significant main effect of treatment on left hind paw stride length (*F*(5, 42) = 0.5929; *p* > 0.05), right hind paw stride length (*F*(5, 42) = 0.4353; *p* > 0.05), or hind paw stride width (*F*(5, 42) = 2.395; *p* > 0.05) (Figure 3A–C). All experimental groups exhibited comparable stride length and stride width values, indicating that neither 6‐OHDA lesioning nor the administration of resveratrol, VitD3, or their combination significantly affected gait parameters under the present experimental conditions.

### 3.3 Dual Neuromotor Assessment Reveals Protective Effects of VitD3 and Resveratrol in Parkinsonian Mice

**FIGURE 4:**
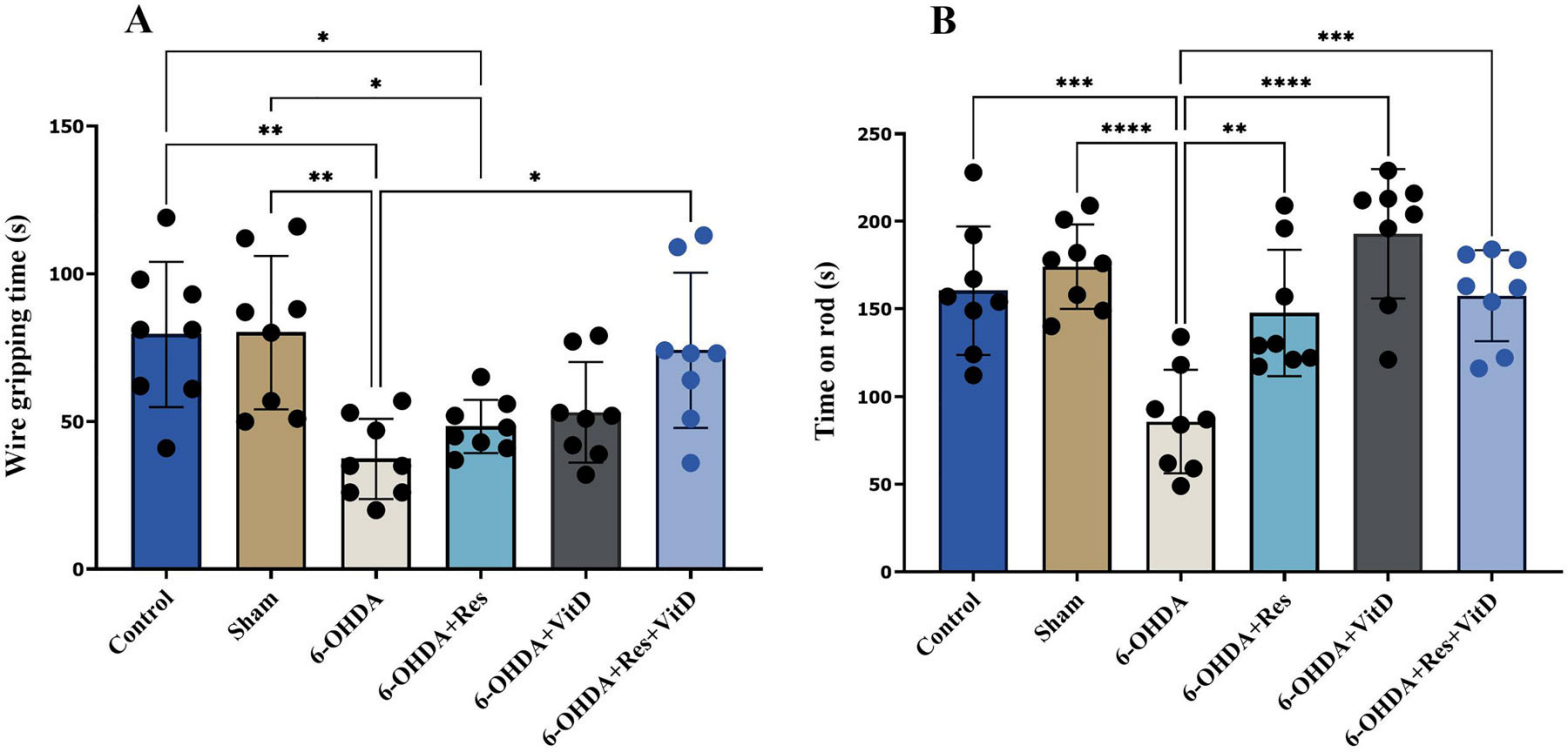
**Muscle strength and motor coordination evaluations**. (A) Wire gripping time in wire grip test and (B) time on rod in rotarod test. Data are expressed as mean ± SEM. 8 animals per group. Statistical comparisons were performed using one‐way ANOVA followed by Tukey's post hoc test. *p < 0.05, **p < 0.01, ***p < 0.001, ****p < 0.0001.

To evaluate muscle strength and coordination, the wire grip test was performed. One‐way ANOVA revealed a significant main effect of treatment on wire gripping time (*F*(5, 42) = 6.301). The 6‐OHDA‐lesioned group exhibited a marked reduction in grip endurance compared with both the sham and control groups (*p* < 0.01). In 6‐OHDA‐treated animals, administration of resveratrol in combination with VitD3, significantly improved wire gripping duration compared to the group treated with 6‐OHDA alone (*p* < 0.05). In 6‐OHDA‐treated animals, monotherapy with either resveratrol or VitD3 alone resulted in a trend toward improvement relative to 6‐OHDA; however, statistical significance was not achieved for this condition (Figure 4A).

Motor coordination and balance were assessed using the rotarod test, which showed a robust overall effect of treatment (*F*(5, 42) = 10.40). The 6‐OHDA group demonstrated a significant decrease in time on the rotating rod when compared with control (*p* < 0.001) and sham animals (*p* < 0.0001). In 6‐OHDA‐treated animals, treatment with resveratrol alone or VitD3 alone markedly prolonged latency to fall (*p* < 0.0001 for VitD3; *p* < 0.01 for resveratrol vs. 6‐OHDA), restoring performance close to control levels. The combination of resveratrol and VitD3 also showed significantly improved motor balance (*p* < 0.001) (Figure 4B).

### 3.4 Combined VitD3 and Resveratrol Normalized *Sirt1* and *Lingo‐1* Gene Expression

**FIGURE 5:**
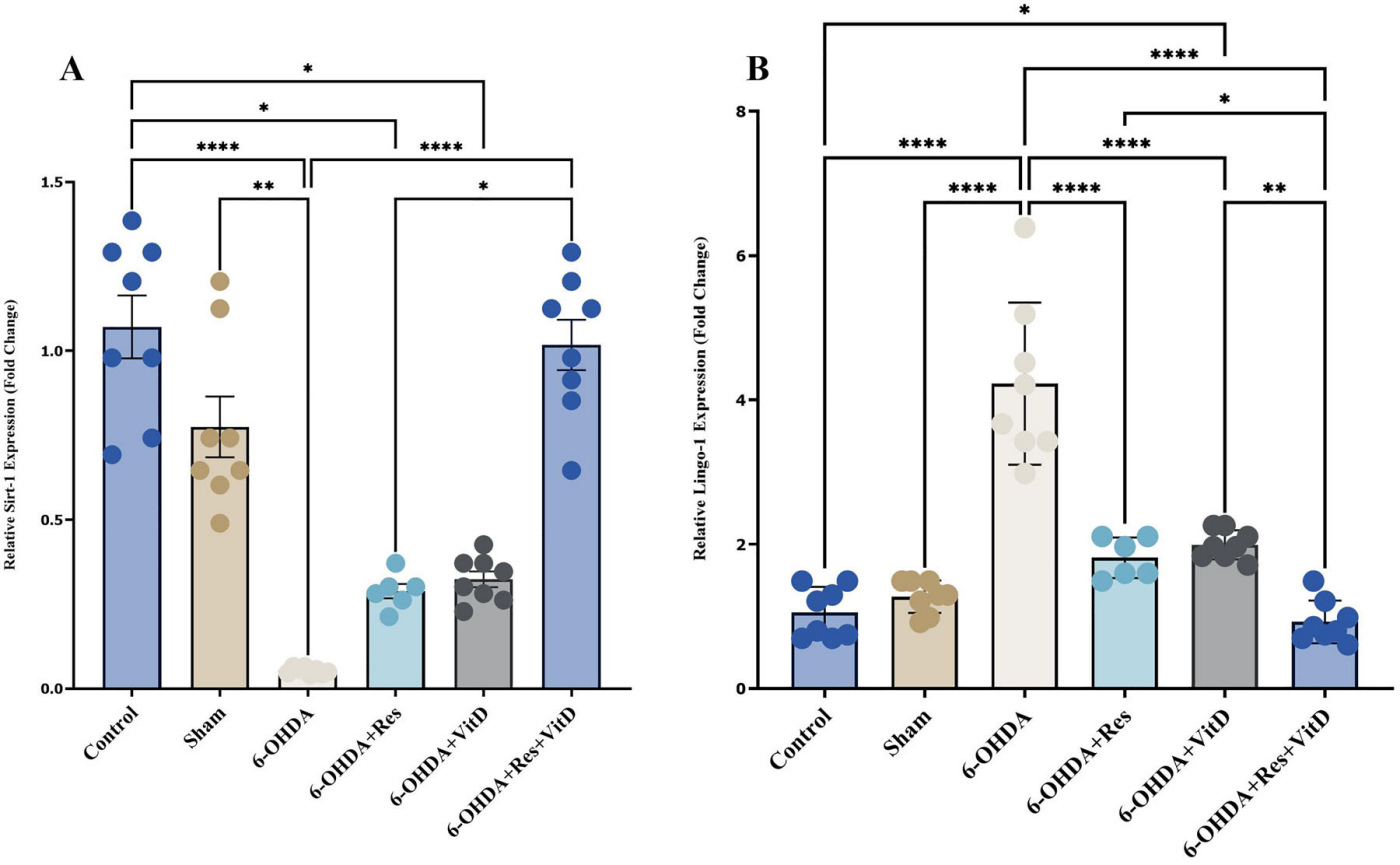
**Ventral tegmental area (VTA) relative gene expression analysis using reverse transcription‐quantitative polymerase chain reaction (RT‐qPCR)**. (A) *Sirt1* and (B) *Lingo‐1*. Data are expressed as mean ± SEM. Statistical comparisons were performed using Kruskall–Wallis followed by Dunn's test for *Sirt1*, and one‐way ANOVA followed by Tukey's post hoc test for *Lingo‐1*. *p < 0.05, **p < 0.01, ***p < 0.001, ****p < 0.0001.

*Sirt1* expression showed marked changes with treatment (*p* < 0.0001 overall effect). The 6 OHDA‐lesioned group exhibited significantly lower levels of *Sirt1* expression compared to control (*p* < 0.001) and sham (*p* < 0.05). Resveratrol or VitD3 alone did not affect *Sirt1* expression, whereas, notably, the combined therapy restored levels to near‐control values (*p* < 0.001 vs. 6‐OHDA alone) (Figure 5A).

*Lingo‐1* was also significantly influenced by treatment (*F*(5, 28)  =  28.88, *p* < 0.0001). The 6‐OHDA group displayed a significantly greater expression of *Lingo‐1* compared to control and sham groups (*p* < 0.0001). Both monotherapies significantly reduced *Lingo‐1* expression when compared to the 6‐OHDA group (*p* < 0.0001). The combined resveratrol +VitD3 regimen also produced a significantly reduced *Lingo‐1* expression compared to the 6‐OHDA group (*p* < 0.0001), as well as the monotherapy groups (Figure 5B).

### 3.5 Ventral Tegmental Area Neuronal Integrity

**FIGURE 6:**
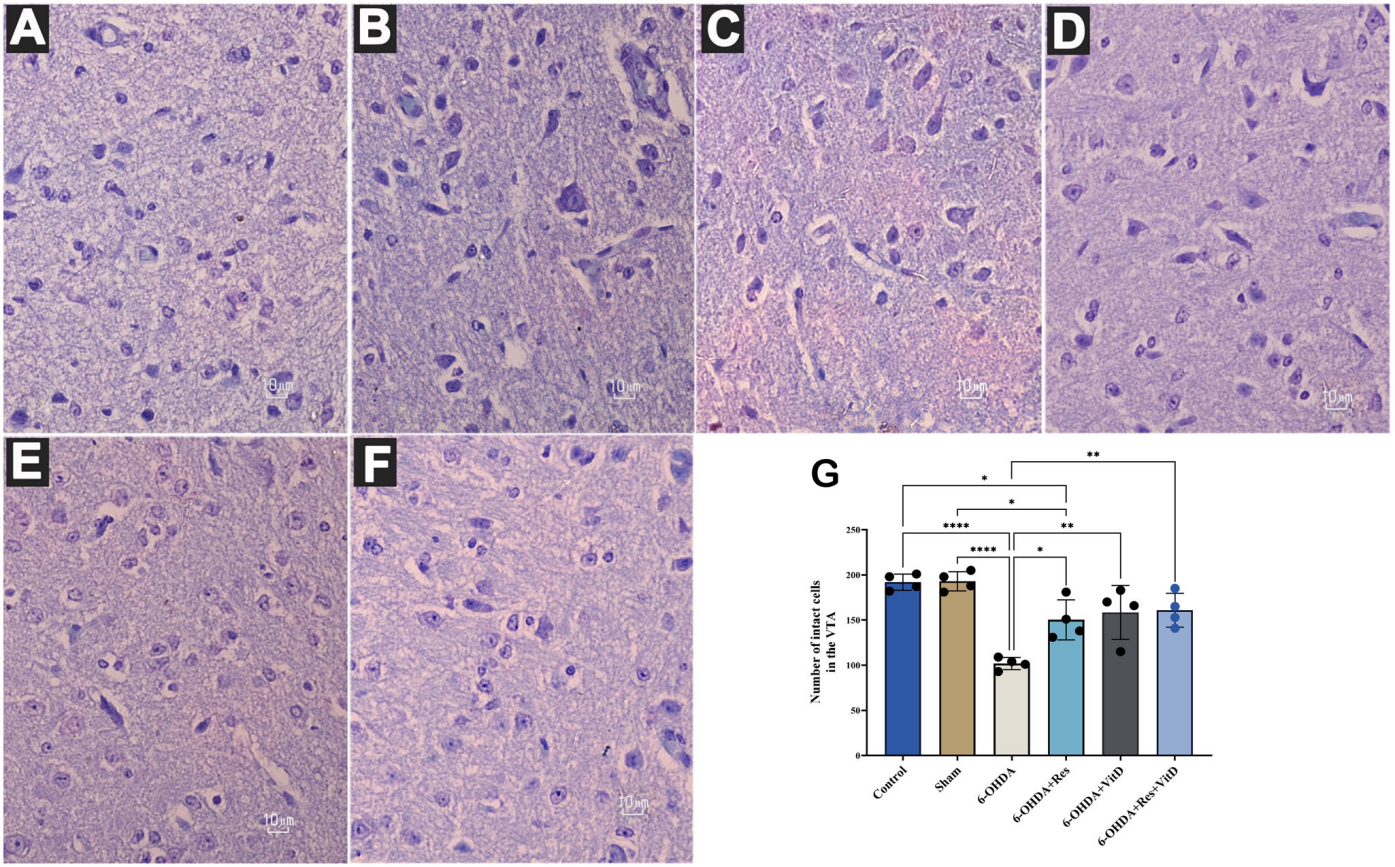
**Nissl staining of the ventral tegmental area (VTA) and quantification of neuronal integrity**. Representative photomicrographs of Nissl‐stained VTA sections from (A) Control, (B) Sham, (C) 6‐OHDA, (D) 6‐OHDA + Res, (E) 6‐OHDA + VitD, and (F) 6‐OHDA + Res + VitD groups. (G) Quantitative analysis of intact neurons per field in the VTA. Data are shown as mean ± SEM; *n* = 4 animals per group. Statistical comparisons were performed using one‐way ANOVA followed by Tukey's post hoc test. *p < 0.05, **p < 0.01, ****p < 0.0001. Scale bar = 10 µm.

Nissl‐stained analysis of the VTA demonstrated significant neuronal loss following 6‐OHDA lesioning (*F*(5, 18)  =  13.63, *p* < 0.0001). Control (Figure 6A) and sham‐operated (Figure 6B) samples contained densely packed, polygonal neurons with prominent Nissl substance, reflecting intact cellular architecture. In contrast, the 6‐OHDA group (Figure 6C) exhibited a marked reduction in neuronal density, shrunken somas, and reduced Nissl staining indicative of neuronal degeneration (*p* < 0.0001 vs. control and sham). Monotherapy treatment with resveratrol (*p* < 0.05; Figure 6D) and VitD3 (*p* < 0.01; Figure 6E) resulted in significantly higher neuronal counts when compared to numbers of intact cells in the 6‐OHDA‐treated group. The combined regimen resulted in significantly greater numbers of surviving cells when compared to the 6‐OHDA‐treated group (*p* < 0.01; Figure 6F) remarkably approaching control and sham values (Figure 6G).

## 4 Discussion

This study provides preliminary evidence that combined administration of VitD3 and resveratrol exerts synergistic neuroprotective effects against 6‐OHDA‐induced Parkinsonian‐like neurodegeneration. The results demonstrate that this combination significantly improves motor performance, which was associated with restoration of *Sirt1* expression, suppression of *Lingo‐1* upregulation, and preservation of neuronal integrity in the VTA. Together, these findings indicate that targeting both *Sirt1* and *Lingo‐1* may underlie the observed behavioral and histological protection, which provides a mechanistic basis for examination of nutraceutical approaches that include VitD3 and resveratrol as disease‐modifying interventions.

The marked motor impairments induced by 6‐OHDA replicate classical Parkinsonian phenotypes such as hypokinesia, impaired coordination, and reduced grip strength, consistent with established characterizations of this model (Soti et al. 2022; Jolicoeur et al. 1991). The restoration of locomotor activity, coordination, and balance following VitD3 and resveratrol treatment suggests the functional relevance of the histological findings. Combined treatment of these two agents resulted in improvements in open field mobility duration, wire grip performance, and rotarod latency, which potentially indicate a preservation of dopaminergic control over basal ganglia and motor cortex output circuits. These behavioral outcomes likely reflect enhanced dopaminergic neurotransmission within the nigrostriatal pathway, but also within the mesocorticolimbic pathway. This is supported by evidence linking the VTA to motor and motivational regulation, demonstrating that dopaminergic projections from this nucleus contribute to fine‐tuned movement coordination and cortical integration (Yetnikoff et al. 2014; Hosp et al. 2019).

Previous research has shown that VitD3 mitigates dopaminergic neurotoxicity through reduction of reactive oxygen species, suppression of neuroinflammation, and modulation of calcium‐dependent apoptotic cascades (Bayo‐Olugbami et al. 2022; Lima et al. 2018; Wang et al. 2001). Resveratrol, in turn, promotes neuronal survival through pleiotropic mechanisms involving mitochondrial preservation, antioxidant defense, and anti‐inflammatory regulation (Schimith et al. 2022). Importantly, resveratrol acts through *Sirt1* activation and downstream deacetylation of PGC‐1α and FOXO3a, enhancing mitochondrial biogenesis and oxidative stress resistance (Lagouge et al. 2006; Sin et al. 2014; Pallas et al. 2009). It also inhibits NF‐κB‐mediated microglial activation and caspase‐3‐dependent apoptosis, contributing to dopaminergic neuron survival in 6‐OHDA and MPTP models (Schimith et al. 2022). Through these integrated pathways, resveratrol restores striatal dopamine levels and improves motor function, reinforcing its therapeutic value as a multi‐target modulator of oxidative, metabolic, and inflammatory stress in PD.

The synergistic neuroprotective effects observed in our study following combined VitD3 and resveratrol treatment may be explained by their cooperative modulation of the vitamin D receptor (VDR) signaling pathway. Resveratrol enhances the transcriptional activity of VDR by promoting its heterodimerization with retinoid X receptor (RXR) and facilitates ligand binding of VitD3 to the receptor (Batie et al. 2015). This cooperative interaction results in increased VDR‐mediated gene transactivation at vitamin D response elements, amplifying downstream effects on antioxidant defense, mitochondrial biogenesis, and cellular survival. Mechanistically, resveratrol also activates *Sirt1*, which is known to modulate VDR activity through chromatin remodeling and coactivator recruitment, thereby reinforcing VitD3‐dependent transcriptional responses (Batie et al. 2015). In the context of PD, this molecular synergy likely enhances dopaminergic resilience by integrating VitD3's genomic neurotrophic effects with resveratrol's *Sirt1*‐mediated mitochondrial protection and anti‐inflammatory actions. Thus, the interaction between resveratrol and VitD3 represents a convergent nutrigenomic mechanism, optimizing VDR signaling and potentiating neuroprotection through coordinated regulation of oxidative stress, energy metabolism, and neuronal survival pathways. Our previous study in the harmaline‐induced essential tremor model provides direct evidence supporting the synergistic interaction between resveratrol and VitD3 (Pirmoradi et al. 2024). Synergistic effects have also been reported in other models like the SAMP8 model of Alzheimer's disease (Cheng et al. 2017). Taken together, multiple studies highlight the therapeutic promise of concurrent resveratrol and VitD3 administration as an approach capable of modulating shared molecular networks implicated in PD and other neurodegenerative disorders.

At the molecular level, *Sirt1* emerged as a potential central mediator of neuroprotection in the VTA in our study. Reduced *Sirt1* expression following 6‐OHDA administration aligns with earlier findings in PD models and human tissue. However, ours is the first study to show that the combination enhances *Sirt1* in the VTA in the 6‐OHDA PD model. *Sirt1* deficiency in PD correlates with heightened oxidative vulnerability and neuronal apoptosis (Singh et al. 2017; Zhu et al. 2021), due to loss of *Sirt1* regulation of mitochondrial biogenesis, antioxidant enzyme synthesis, and suppression of pro‐apoptotic signaling (Ou et al. 2014; Liang et al. 2020; Lee and Gu 2013). In our study, the combination of resveratrol and VitD3 restored *Sirt1* expression to control levels in the VTA, likely due to complementary mechanisms converging on *Sirt1* regulation. Resveratrol directly activates *Sirt1* by increasing intracellular NAD^+^ availability and enhancing its deacetylase activity, while VitD3 upregulates *Sirt1* transcription through VDR‐mediated genomic pathways (Yang et al. 2025; Manna et al. 2017; Talib et al. 2023). Therefore, the cooperative engagement of these metabolic and transcriptional mechanisms likely amplifies *Sirt1* signaling efficiency, leading to normalization of its expression despite 6‐OHDA‐induced downregulation.

Parallel to *Sirt1* activation, the suppression of *Lingo‐1* expression observed in this study reveals another major neuroprotective mechanism. *Lingo‐1* functions as a negative regulator of axonal growth and myelination. Overexpression of *Lingo‐1* has been observed in PD models, where it contributes to axonal degeneration and failure of dopaminergic regeneration (Inoue et al. 2007). Moreover, *Lingo‐1* genetic variants are known to be associated with PD (Vilariño‐Güell et al. 2010). In the present study, VitD3 and resveratrol each reduced *Lingo‐1* expression, with the combination leading to a greater suppression than that seen with monotherapies alone. This finding aligns with our previous report in essential tremor, where we observed that the combination therapy of resveratrol and VitD3 resulted in normalization of *Lingo‐1* overexpression (Pirmoradi et al. 2024).

Histological findings of our study corroborate the behavioral and molecular data, showing significant preservation of VTA neurons following the combination treatment. The VTA, which plays a key role in motivation and motor control, is increasingly recognized as vulnerable in PD due to its high metabolic demand and sensitivity to oxidative damage (Alberico et al. 2015). The preserved neuronal morphology and reduced degeneration observed here likely reflect the combined effects of *Sirt1* activation and *Lingo‐1* inhibition, resulting in enhanced mitochondrial efficiency and axonal integrity.

**FIGURE 7:**
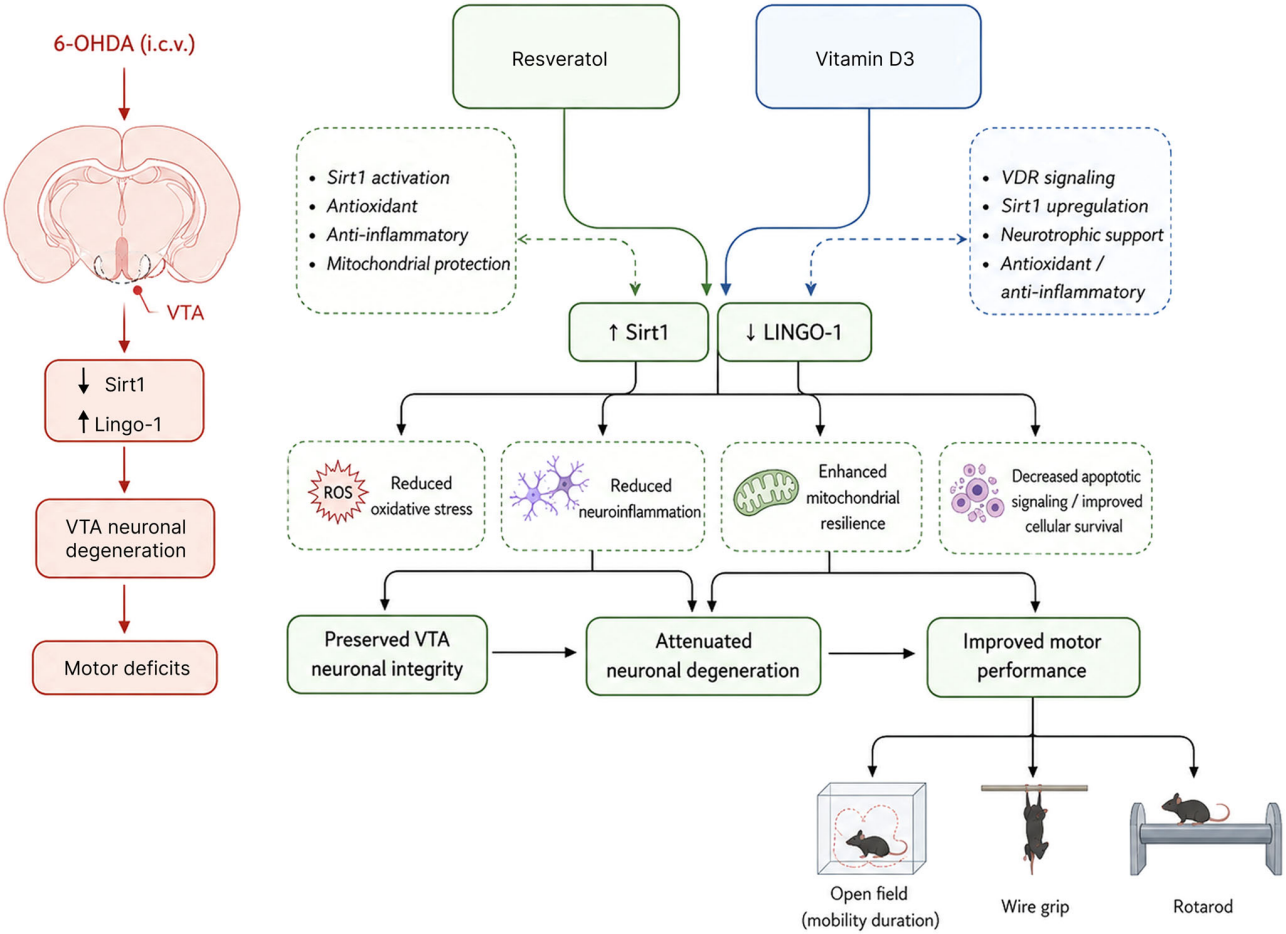
**Proposed neuroprotective mechanism of resveratrol and vitamin D3 in 6‐OHDA‐induced Parkinsonism**. 6‐OHDA induces oxidative stress, neuroinflammation, *Sirt1* downregulation, *Lingo‐1* upregulation, VTA neuronal degeneration, and motor impairment. Resveratrol and vitamin D3 are proposed to act through enhancement of *Sirt1* signaling and suppression of *Lingo‐1*, thereby reducing oxidative and inflammatory injury, improving mitochondrial resilience, preserving VTA neuronal integrity, and improving motor performance.

Taken together, our findings support an integrative neuroprotective model in which VitD3 and resveratrol synergistically activate *Sirt1* while suppressing *Lingo‐1*. These molecular cascades translate into histological protection of neurons and behavioral recovery, representing a promising avenue for disease‐modifying strategies in PD. An overview of proposed neuroprotective mechanisms of resveratrol and vitD3 against 6‐OHDA is depicted in Figure 7.

Nonetheless, several limitations of our study warrant further investigation. To minimize the variation due to estrous cycle and sex hormone fluctuations, only male mice were used in the present study. Therefore, the results should be regarded as pertinent only to male mice and further work in females is needed to determine whether sex‐dependent differences impact the neuroprotective effects of combined vitamin D3 and resveratrol treatment. Second, while the doses of vitamin D3 and resveratrol were chosen based on previous experimental studies showing efficacy and safety, their concentrations in plasma and brain were not measured. Thus, bioavailability and tissue accumulation of these compounds could not be directly assessed. A further support to the interpretation of the therapeutic effects observed could be future studies including pharmacokinetic analyses (e.g., LC–MS/MS). Third, the assessment of neuronal survival was performed by Nissl staining, which contains useful information about neuronal morphology and cell loss, but does not specifically identify dopaminergic neurons. Future investigations should incorporate tyrosine hydroxylase (TH) immunohistochemistry to more specifically quantify dopaminergic neuronal preservation. In addition, although the 6‐OHDA model effectively reproduces dopaminergic neurodegeneration, it does not fully recapitulate the progressive or multisystemic aspects of PD. Additional studies using chronic or α‐synuclein‐based models could provide further insight into long‐term neuroprotective mechanisms. Additionally, evaluation of other underlying mechanisms, including RhoA phosphorylation, AMPK activity, and Nrf2 nuclear localization would clarify the signaling hierarchy downstream of *Sirt1/Lingo‐1* modulation. Moreover, the precise molecular interactions between VitD3, resveratrol, and the VDR–*Sirt1* pathway remain to be delineated in dopaminergic neurons, as most existing evidence is derived from peripheral or cancer cell lines. Addressing these limitations will be crucial to establish the therapeutic potential and mechanistic depth of combined VitD3 and resveratrol in neurodegenerative diseases.

## 5 Conclusions

In conclusion, combined VitD3 and resveratrol therapy exerts neuroprotective effects against 6‐OHDA‐induced dopaminergic degeneration. The combination therapy resulted in activation of *Sirt1* and suppression of *Lingo‐1*, which was associated with improved motor outcomes and preservation of VTA neurons. These findings provide a mechanistic basis for developing combinatorial nutraceutical therapies targeting *Sirt1* and *Lingo‐1* expression as a novel disease‐modifying approach for PD.

## Author Contributions

**Monavareh Soti**: conceptualization, methodology, investigation, data curation, formal analysis. **Mehran Ilaghi**: conceptualization, methodology, investigation, writing – original draft. Kristi A. Kohlmeier: writing ‐ review and editing. **Erfan Shahabinejad**: conceptualization, methodology, writing – original draft, writing – review and editing. **Zeynab Pirmoradi**: conceptualization, methodology, formal analysis, supervision, project administration, writing – review and editing. **Mohammad Shabani**: conceptualization, formal analysis, resources, supervision, funding acquisition, writing – review and editing.

## Funding

Funding for this study was provided by Torbat Heydariyeh University of Medical Sciences (Grant no. 404000061).

## Conflicts of Interest

The authors declare that no competing and financial interests exist.

## Ethics Statement

All study protocols have been conducted under approval of the Ethics Committee of Torbat Heydariyeh University of Medical Sciences (Ethics approval code: IR.THUMS.AEC.1405.001).

## Data Availability

The datasets used or analyzed during the current study are available from the corresponding author upon request.
